# Spatial Pattern Analysis of Heavy Metals in Beijing Agricultural Soils Based on Spatial Autocorrelation Statistics

**DOI:** 10.3390/ijerph8062074

**Published:** 2011-06-08

**Authors:** Xiao-Ni Huo, Wei-Wei Zhang, Dan-Feng Sun, Hong Li, Lian-Di Zhou, Bao-Guo Li

**Affiliations:** 1 Beijing Academy of Agriculture and Forestry, Beijing 100097, China; E-Mails: hxnsky@126.com (X.-N.H.); Zhangwei492@163.com (W.-W.Z.); liandizhou@126.com (L.-D.Z.); 2 Department of Land Resources and Management, College of Natural Resources and Environment Science, China Agricultural University, Beijing 100193, China; E-Mails: sundf@cau.edu.cn (D.-F.S.); libg@cau.edu.cn (B.-G.L.); 3 Department of Environmental Engineering, Taiyuan University, Taiyuan 030009, China

**Keywords:** heavy metals, spatial pattern, Moran’s I statistic, Beijing agricultural soils

## Abstract

This study explored the spatial pattern of heavy metals in Beijing agricultural soils using Moran’s I statistic of spatial autocorrelation. The global Moran’s I result showed that the spatial dependence of Cr, Ni, Zn, and Hg changed with different spatial weight matrixes, and they had significant and positive global spatial correlations based on distance weight. The spatial dependence of the four metals was scale-dependent on distance, but these scale effects existed within a threshold distance of 13 km, 32 km, 50 km, and 29 km, respectively for Cr, Ni, Zn, and Hg. The maximal spatial positive correlation range was 57 km, 70 km, 57 km, and 55 km for Cr, Ni, Zn, and Hg, respectively and these were not affected by sampling density. Local spatial autocorrelation analysis detected the locations of spatial clusters and spatial outliers and revealed that the pollution of these four metals occurred in significant High-high spatial clusters, Low-high, or even High-low spatial outliers. Thus, three major areas were identified and should be receiving more attention: the first was the northeast region of Beijing, where Cr, Zn, Ni, and Hg had significant increases. The second was the southeast region of Beijing where wastewater irrigation had strongly changed the content of metals, particularly of Cr and Zn, in soils. The third area was the urban fringe around city, where Hg showed a significant increase.

## 1. Introduction

Heavy metals often accumulate to excess in agricultural soils due to natural processes and anthropogenic activities. Since these can cause adverse effects on the environment, heavy metal soil pollution is an urgent problem. Successful assessment and remediation of heavy metal pollution in soils will depend on the understanding of their spatial variability and the relationships between heavy metals and the factors leading to pollution.

Both geostatistics and spatial autocorrelation statistics (Moran’s I) are the methods used for exploring the spatial pattern of heavy metals. Generally, geostatistics is widely used [1–4], but it cannot determine whether the spatial correlation is positive or negative, and the significance of spatial dependence. In addition, geostatistics cannot detect spatial outliers, whose values obviously different from the values of their surrounding location, can make the semivariogram erratic. Compared with geostatistics, global Moran’s I can be used to estimate the strength and determine the positive or negative of the spatial correlation of a variable, local Moran’s I can be used to identify spatial clusters and spatial outliers of a variable [5–7]. Furthermore, the significance of the spatial correlation can be tested [8,9]. Although spatial autocorrelation was defined decades ago and has been widely used in many studies [8,10,11], only the global Moran’s I statistic has been used for the spatial variability of heavy metals [10]. Reports on analyzing multi-scale spatial variability of heavy metals in soils using the Moran’s I statistic have not yet been published, particularly in relation to the local Moran’s I statistic for understanding the structure of spatial dependence.

For heavy metals in Beijing soils, there are some related studies. However, the emphasis of such studies just covered the urban-rural transition zone, periurban, and rural zones, and/or had a relatively few sampling points [12–14]. Thus, in order to reflect the whole and representative real situation of heavy metals in whole Beijing agricultural soils, Huo *et al*. further assessed the spatial variability of heavy metals with a total of 1,018 samples covering the entire Beijing agricultural soils using geostatistics [15]. Considering the advantages of the Moran’s I statistic, it can help to identify the more characteristics of the spatial pattern of heavy metals, because understanding the spatial pattern is the basis for environmental quality assessment and soil remediation.

Therefore, the main objectives of this research were: (1) to explore the spatial variability of heavy metals in Beijing agricultural soils using the spatial autocorrelation statistic, including the influence of different weight matrixes and sampling density on spatial autocorrelation; and (2) to reveal the local spatial patterns of heavy metals in Beijing agricultural soils in order to identify the potential risk areas for heavy metal pollution and its possible causes.

## 2. Materials and Methods

### 2.1. Study Area

**Figure 1.**
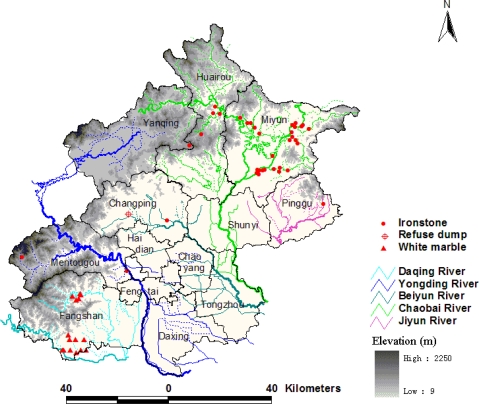
The study area.

Beijing municipality is located in the northwest region of the north China plain, between longitude 115°25′–117°30′E and latitude 39°28′–41°05′N, and covers an estimated area of 1.6 × 10^4^ km^2^. The elevation of Beijing ranges from 2,250 m in the northwest to 9 m in the southeast. Mountains cover approximately 62% of the entire area and plains cover the remaining 38% (Figure 1). The area has a temperate continental monsoonal climate with an average annual temperature of 11.8 °C (average maximum 26 °C in July and average minimum −5 °C in January). The mean annual precipitation in the area is 470–660 mm, with approximately 60% of the precipitation occurring in July and August. The average annual evaporation is 1,800–2,000 mm. The area is the source of five large rivers, the Yongding, Chaobai, Beiyun, Jiyun and Daqing (Figure 1). Average annual runoff is about 1.8 × 10^9^ m^3^ but has decreased to 1.3 × 10^9^ m^3^ since the end of the last century. The Yongding River provides water mainly for industry, the Chaobai River mainly for resident living, and the Beiyun River plays a dual role in wastewater drainage from industry and human living activities.

The primary types of agricultural soil in the area include Cab Ustic Luvisols, Hap Ustic Luvisols and Och Aquic Cambisols. According to the second agricultural census in 2006, cultivated land occupied 2.32 × 10^5^ hm^2^, and orchard land 1.22 × 10^5^ hm^2^, together covering 21% the total area of Beijing. The fertilizer input was 517.5 kg/hm^2^, about 1.44 times of the national average level, and the pesticide inputs were 8.11 kg/hm^2^, or about 2.33 times of the country level. The overuse of fertilizer and pesticides has imposed heavy environmental pressures on the area. The metal mineral resources were primarily Fe, Ag, Zn, and Pb elements, and ironstone was exploited on a large-scale, mainly distributed in the Huairou district and Miyun county (Figure 1). In addition, there had been white marble exploration in southwest Beijing (Figure 1). The mineral resource exploration and industry development in Beijing also had negative environmental effects on the area.

### 2.2. Soil Sampling and Analysis

**Figure 2.**
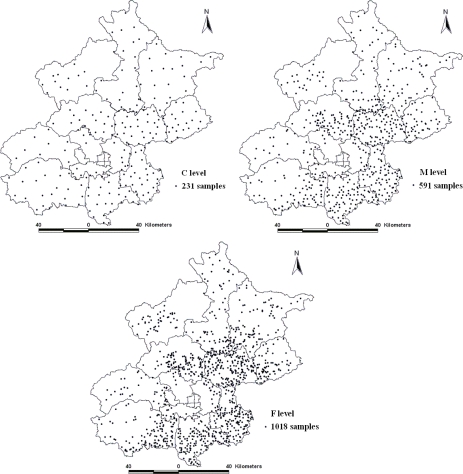
Distribution of soil samples at the three densities.

To investigate the pollution status of heavy metals in Beijing agricultural areas, a large–scale soil sampling project was conducted after the crop harvest in the Autumn of 2006. According to the agricultural land distribution and land use type maps of Beijing, a non-uniform distribution of the stratified sampling technique was adopted to collect samples and ensure the representativeness of sample. The sampling process was divided into three steps to collect a total of 1,018 samples. First, 231 soil samples were collected from the agricultural soils in the entire study area, with uniform sampling being the low sampling density (C). Secondly, another 360 soil samples were added from areas with more agricultural soils to create the medium sampling density (M). Third, 427 soil samples were further collected on the basis of the two previous samplings and the agricultural soils to make a high sampling density (F). General ideal is that more areas of agricultural land more sampling points. Figure 2 shows the distribution of soil samples at the three sampling densities.

For each sample, five surface soil (0∼20 cm) sites were sampled within 10 × 10 m square areas and then mixed. Global Positioning System was used to precisely locate each sampling position (latitude and longitude); and a total of 1 kg of mixed soil per sample was collected. All soil samples were collected using a stainless steel spade and a scoop made from bamboo and then stored in polyethylene bags.

The soil samples were air-dried, crushed in an agate mortar, and then passed through a 100-mesh nylon sieve. The concentrations of eight heavy metals, including Cr, Ni, Cu, Zn, As, Cd, Pb, and Hg, were analyzed in the soil samples following the Chinese Environmental Quality Standard for Soils (GB15618-1995).

### 2.3. Global Spatial Autocorrelation

Spatial autocorrelation is an assessment of the correlation of a variable with reference to its spatial location and it deals with the attributes and the locations of the spatial features [16]. Moran’s I is a popular test statistic for spatial autocorrelation.

Global Moran’s I is a global test statistic for spatial autocorrelation, which is based on cross-products for measuring attribute association. It is calculated for *n* observations on a variable *x* at locations of *i* and *j*, as follows [17]:

(1) $$I=\frac{n}{\sum_{i=1}^{n}\sum_{j=1}^{n}w_{ij}}\cdot \frac{\sum_{i=1}^{n}\sum_{j=1}^{n}w_{ij}\left(x_{i}-\bar{x}\right)\left(x_{j}-\bar{x}\right)}{\sum_{i=1}^{n}{\left(x_{i}-\bar{x}\right)}^{2}}\;\;\;i\neq j$$

where *n* is the number of observations of the whole region, *x_i_* and *x_j_* are the observations at locations of *i* and *j*, *x̄* is the mean of *x*, and *w_ij_*, an element of spatial weights matrix *w*, is the spatial weight between locations of *i* and *j*.

The weight matrix depicts the relationship between an element and its surrounding elements. The weights are based on contiguity relations or distance. In a weight matrix based on contiguity, a value unequal to zero in the matrix represents pairs of elements with a certain contiguity relation and a zero represents pairs without contiguity relation. The rook case and queen case are the typical examples for contiguity relation. The first takes only four neighbours into account with common boundaries, and the latter takes into account all eight surrounding cells, including common boundaries and common corners. In a distance-based weight matrix, a threshold distance is specified such that all locations within the given distance are considered to be “neighbors”. Alternatively, the k-nearest neighbor weight matrix is also based on distance, which is computed as the distance between a point and the number (k) of nearest neighbor points.

The value of Moran’s I will vary between 1 and −1. A higher positive Moran’s I implies that values in neighboring positions tend to cluster, while a low negative Moran’s I indicates that high and low values are interspersed. When Moran’s I is near zero, there is no spatial autocorrelation, meaning that the data are randomly distributed [8,9].

The Moran scatter plot describes the distribution of all observation values (x-axis) in relation to their neighbors (y-axis). Observations in the lower left (Low–low) and upper right (High–high) quadrant represent potential spatial clusters (values surrounded by similar neighbors), whereas observations in the upper left (Low–high) and lower right (High–low) suggest potential spatial outliers (values surrounded by dissimilar neighbors) [18]. The slope of the scatter plot corresponds to the value of global Moran’s I, therefore, the scatter plot can help to visualize the strength of the overall spatial autocorrelation, as well as the complexity of spatial autocorrelation types in the study area. However, the Moran scatter plot does not indicate the significance of spatial autocorrelation types and the locations of the clusters or outliers.

### 2.4. Local Spatial Autocorrelation

Local Moran’s I is a local test statistic for spatial autocorrelation, and identifies the autocorrelation between a single location and its neighbors. It is computed as follows:

(2) $$I_{i}=\frac{n\left(x_{i}-\bar{x}\right)\sum_{j=1}^{n}w_{ij}\left(x_{j}-\bar{x}\right)}{\sum_{i=1}^{n}{\left(x_{i}-\bar{x}\right)}^{2}}$$

The notations in Equation (2) are as described for Equation (1), but the corresponding values are from the local neighboring region.

A map showing locations with significant Local Moran statistics, classified by types of spatial correlation, is defined as a Local Indicators of Spatial Autocorrelation (LISA) cluster map [5]. Four categories of local spatial autocorrelation are distinguished; two of these suggest clusters and two of these suggest outliers. The LISA map provides information about which clusters/outliers are statistically significant and indicates the variability and distribution of the spatial correlation types in the study area. Therefore, LISA is an important tool for detecting “interesting” locations and assessing the extent to which the spatial distribution exhibits “spatial heterogeneity” [5].

### 2.5. Data Treatment with Computer Software

Soil samples were stored using the ArcView 3.2 software to create a spatial database. The Thiessen polygons of samples were created and all spatial analysis including global and local spatial autocorrelation was carried out using Geoda095i software [18].

## 3. Results and Discussion

### 3.1. Influence of Different Weight Matrixes on Spatial Autocorrelation

Eight different spatial weight matrixes were used to impose a neighborhood structure on the 1,018 samples and assess the spatial autocorrelation of eight heavy metals. The results of global Moran’s I values are given in Table 1. The significance of Moran’s I were tested (*p* < 0.05). Cr, Ni, Zn, As, Cd, and Hg showed significant and positive spatial correlations on all spatial weights, but As and Cd had low Moran’s I values close to 0. There were no significant spatial correlations for Cu on any spatial weights. Except for the 4-nearest neighbors weight, Pb had spatial significant correlations on other types of weights, but the values of Moran’s I were near 0.

Generally, the numbers of neighbours using the queen criterion will be equal to or greater than that using the rook criterion. However, Table 1 shows that the global Moran’s I values based on the first order rook and queen weights were equivalent. This may be because they had the same connectivity histogram. This indicated that the direct neighboring relations were not affected by the direction of four neighbors or eight neighbors. Spatial autocorrelation coefficients generally decreased with the increase of the number (k) of nearest neighbor points, following the rule of the farther the distance, the less attribute similarity (Table 1). The metals had relatively high Moran’s I values with the 4 km distance band spatial weight matrix. Furthermore, for irregular samples, the reasonable weight matrix is the distance-based weight matrix. Therefore, the subsequent spatial correlation analysis was calculated using this weight matrix based on distance, and focused on the four elements, Cr, Ni, Zn, and Hg, which had significant and positive spatial correlations.

Because the selection of spatial weigh was empirical, as well as the same weight matrix under a certain distance limit was assigned to all points, there may be had a certain impact on the spatial autocorrelation of the heavy metals. If the spatial weights based on decay distance were designed, the results of the influence of spatial weight on spatial autocorrelation of heavy metals may be more reasonable.

**Figure 3.**
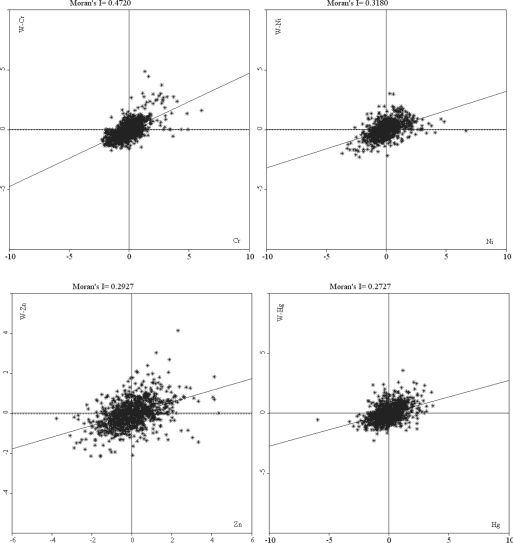
Moran scatter plot for Cr, Ni, Zn, and Hg.

Figure 3 shows the Moran scatter plots of Cr, Ni, Zn, and Hg with 1,018 samples, in which the horizontal axis was the standardized value of heavy metal concentration and the vertical axis was the standardized value of the neighboring heavy metal concentration. A large part of the samples of the four metal elements mainly clustered in the left lower and right upper quadrants, indicating that a positive spatial autocorrelation dominated the overall spatial pattern.

There was also a certain part of the samples in the right lower and left upper quadrants, indicating that negative spatial autocorrelation could not be neglected. With the decrease in spatial autocorrelation coefficients, the scatter plot tended to became further disaggregated, and these samples were far from the Moran’s I regression line and strongly influenced the global spatial autocorrelation, particularly for Cr and Zn, indicating some local nonstationarity (Figure 3). Consequently, the variability of their spatial patterns should be considered.

### 3.2. The Effect of Sampling Density on Spatial Autocorrelation

**Figure 4.**
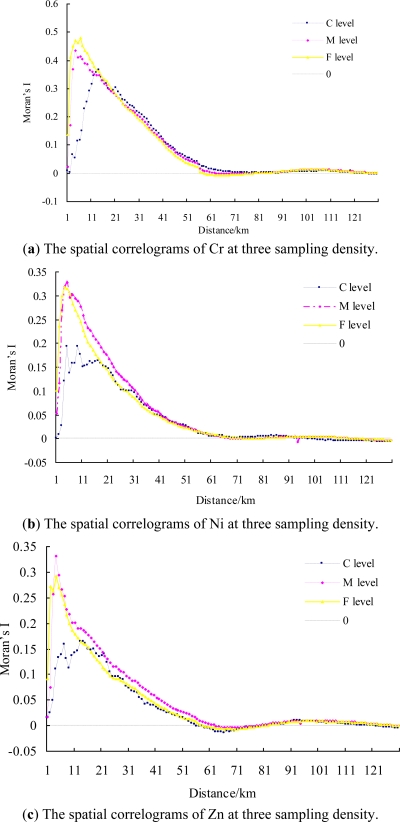

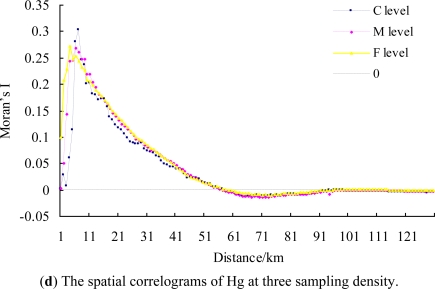
The spatial correlograms of the metals at three sampling density.

Moran’s I values can be plotted against distance classes, called a spatial correlogram [8]. Figure 4 gives the spatial correlograms for Cr, Ni, Zn, and Hg produced with a weight matrix based on distance at three sampling densities. The Moran’s I of Cr, Ni, Zn, and Hg all initially increased as a peak with the increase of distance, then dropped down to 0 with further increase of the distance at three density levels. This revealed, at all three sampling densities, the four heavy metals displayed stronger spatial dependence initially as the distance expanded to include more close points, whereas their spatial dependence decreased as the distance further increased to include more distant points. Normally, the distance where the 0 value first appears is considered as the maximal spatial positive correlation range, which was about 57 km, 70 km, 57 km, and 55 km for Cr, Ni, Zn, and Hg, respectively (Figure 4).

Moran’s I for Cr had a distinct difference within 13 km, then the difference disappeared for the three density levels [Figure 4(a)]. Similarly, Ni, Zn, and Hg also had significant differences when the distance was less than 32 km, 50 km, and 29 km, respectively [Figure 4(b), (c), (d)]. This indicated that sampling density had an effect on the spatial dependence, but the effect was no longer obvious beyond a certain distance. Consequently, with the increase of the distance, the far neighbors gradually imposed on the spatial dependence instead of the sampling density, and sampling density had no influence on the maximal spatial positive correlation ranges of the four metals.

Figure 4(a) shows that the peak of the spatial correlogram for Cr was highest at the F level at about 6 km characteristic distance. As the sampling density decreased, the peak decreased, but the distance increased as the peak became greater. For Zn and Ni, the peaks at the F and M level were close about 4 km, and somewhat higher at the M level, while that at the C level was significantly low and fluctuant [Figure 4(b), (c)]. For Hg, the highest peak was at the C level about 6 km, and the peaks at the F, M level were about 4 km, but the peak values were relatively low [Figure 4(d)]. Therefore, Cr should adopt a high-density level, Hg can adopt a low-density level, and Zn and Ni can use a medium-density level for global spatial autocorrelation analysis, in order to reduce the sample numbers. In addition, the distances in which the peaks appeared at three sampling densities should be adopted for the spatial dependence analysis.

The spatial correlogram analysis revealed that Cr, Ni, and Zn had the similar sampling density effect. For Cr, Ni, and Zn, the higher sampling density enhanced the spatial dependence. In contrast, it may be the existence of extreme value, the spatial dependence of Hg at higher sampling density became weaker. These scale effects existed within a certain distance. Moreover, the spatial correlogram can help to find the maximal spatial positive correlation range and the suitable neighborhoods for spatial dependence analysis. Therefore, LISA was further used to identify the detail spatial variability for the four metals at the F density level.

### 3.3. Local Indicators of Spatial Association (LISA)

The LISA indicates the spatial variability details. The distance where the spatial dependence of Cr, Ni, Zn, and Hg was the strongest at F density level is selected to reveal the local spatial pattern (High-high, Low-low, High-low, Low-high and no significance). The distance weight matrix was 6 km, 4 km, 4 km, and 4 km for Cr, Ni, Zn, and Hg, respectively.

Although the four heavy metals had a significant global spatial positive correlation, more than half of the samples of the four metals had no significant spatial pattern (Table 2). Table 2 shows 36.5% Cr, about 20% of the other metals samples belonged to the significant spatial clusters, which were the largest spatial pattern. As the trace elements in soils, Low-low pattern of the four heavy metals dominated the overall spatial pattern, as well as High-high patterns that were more than half that of the Low-low pattern. About 10% samples of the metals also were significant spatial outliers, and the Low-high pattern for Ni, Zn, and Hg was overwhelming (Table 2). These significant spatial patterns can indicate strong ongoing enrichment processes of the four heavy metals in Beijing agricultural soils.

The samples with Cr pollution were only 0.69% and all of these occurred in the significant High-high spatial pattern. Zn pollution was less (only 0.1% samples) in the no significant spatial pattern type (Table 3). Hg and Ni represented a relatively high percentage of polluted samples, and about 6.2% of the samples were polluted by Hg. Among these, 2.26% of the samples were in significant High-high spatial clusters, 0.49% in significant Low-high, and only 0.10% in significant High-low spatial outliers (Table 3). For Ni, about 3.93% of the samples were polluted and 0.79% of the samples were in significant High-high spatial clusters, and 0.49% in significant Low-high spatial outliers (Table 3).

Compared with the spatial randomness, the significant spatial patterns of these heavy metals demonstrated that the underlying enrichment processes were more stable, and as such, would present more difficulties for their remediation. In addition, in soil heavy metal evaluation, the outliers may represent the potential pollution areas, such as Ni and Hg in this study. If further spatial interpolation will be produced, the outliers cannot be deleted arbitrarily and should adopt more complicated geostatistics approach.

**Figure 5.**
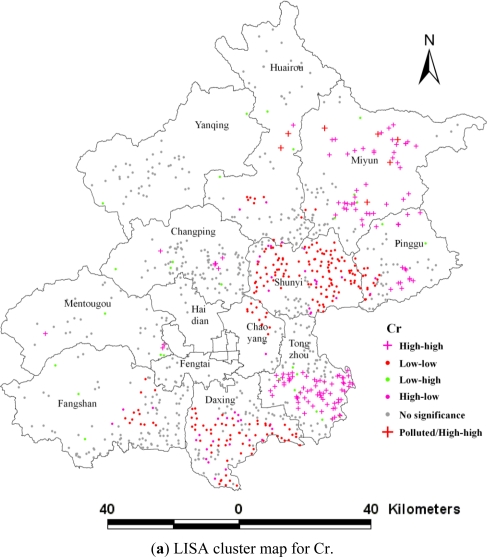

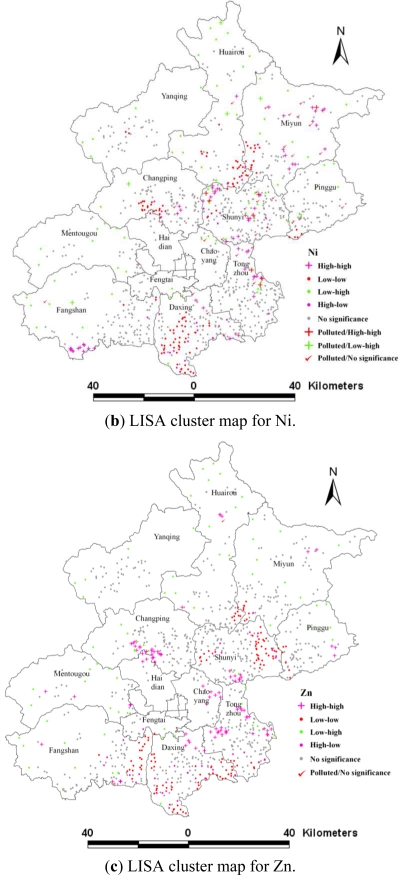

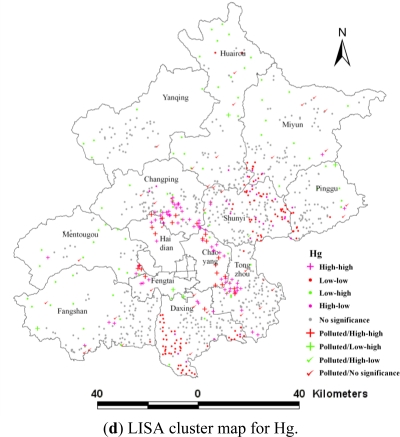
LISA cluster maps for heavy metals.

The LISA map can further detect the locations of the interesting spatial patterns for heavy metals. The northeast region was strongly influenced by High-high pattern of Cr, Ni [Figure 5(a), (b)], where some iron mines are distributed (Figure 1). The second hot region was the southeast Beijing, in which Cr, Ni, and Zn displayed a significant High-high pattern [Figure 5(a), (c)]. Moreover, High-low outliers of the three metals were mainly distributed in this region, and were found near Low-low clusters, indicating that anthropogenic activities had begun to change the Low-low pattern. In areas downstream of Beijing city, there had been long-term wastewater irrigation history in southeast Beijing, which led to heavy metal contamination [19]. The third interesting area was the urban fringe, particularly the northern and eastern parts, where clusters of High-high Hg were covered [Figure 5(d)]. Emissions from human activities such as the combustion of fossil fuels, the burning of coal, industrial boilers, and petroleum refineries had resulted in significant increases in the emission of Hg in and around urban areas [20,21]. As well, high concentrations of Cr, Ni, and Zn were clustered in and around the refuse dump in the southern Changping district, where landfill and waste incineration were the potential pollution sources (Figure 1, Figure 5).

However, because the LISA map was generated based on the soil samples, the boundaries between different spatial pattern types are easily confused and unreadable. In future research, the method of zoning should be introduced for improving to distinguish the spatial pattern types of boundaries. Such as, LISA clusters map, with other possible driving factors maps in GIS software, can also quantify their spatial relationships to confirm and refine their effects. Their distributions could be used to delineate the potential monitoring and remediation zones. Moreover, these zones can assist in developing measures and policies that can be responsive to the spatial variations and pollution processes.

## 4. Conclusions

Compared with geostatistics, although spatial autocorrelation analysis cannot be used to estimate unobserved points, global and local Moran’s I analysis have their advantages. The strength and significance of spatial dependence could be easily compared and tested. Moreover, the spatial correlogram can describe the changes of spatial dependence with distance, which indicate the maximal spatial positive correlation range and the suitable neighborhoods for spatial dependence analysis. In addition, local Moran’s I analysis can help to detect spatial outliers or hot spots. Therefore, it is an effective exploratory spatial analysis technique for regional variables.

This study explored spatial pattern of heavy metals in the entire Beijing agricultural soils based on 1,018 samples collected in 2006 using Moran’s I analysis. The four elements Cr, Ni, Zn, and Hg had significant and positive spatial correlations with large Moran’s I values. The Moran’s I values were affected by spatial weight matrix. Cr, Ni, and Zn had similar sampling density effects. Higher sampling density enhance the spatial dependence, whereas, higher sampling density may reduce the spatial dependence for Hg. In addition, the maximal spatial positive correlation ranges of the four metals did not change at different sampling density. The global Moran’s I of the four metals was scale-dependent on distance, initially taking stronger spatial dependence with the increase of the distance, then becoming weaker with further expansion of the distance. It is worth noting that the scale effect existed in a certain distance.

The local spatial autocorrelation analysis revealed that the four metals all had important High-high pattern, and Low-high and High-low spatial outliers, indicating strong enrichment processes for the four heavy metals in Beijing agricultural soils. Thus, these areas should be receiving more attention: the northeast and southeast region of Beijing, where significant increases in Cr, Zn, Ni, and Hg occurred, as well as the urban fringe around city where Hg showed a significant increase. The global Moran’s I was proved to be a useful measure of overall clustering, while the local Moran’s I was an important tool for detecting local spatial patterns for possible polluting areas or interesting patterns for further monitoring. Therefore, spatial autocorrelation analysis can be a useful method to explore the spatial pattern of heavy metals.

## Figures and Tables

**Table 1.** Global spatial autocorrelation coefficient (global Moran’s I value) based on different spatial weighs for heavy metals.

**Spatial weights**	**Cr**	**Ni**	**Cu**	**Zn**	**As**	**Cd**	**Pb**	**Hg**
First order rook contiguity	0.495 *	0.317 *	0.026	0.282 *	0.077 *	0.119 *	0.051 *	0.290 *
First order queen contiguity	0.495 *	0.317 *	0.026	0.282 *	0.077 *	0.119 *	0.051 *	0.290 *
4-nearest neighbors	0.541 *	0.327 *	0.006	0.287 *	0.071 *	0.124 *	0.036	0.283 *
5-nearest neighbors	0.521 *	0.320 *	0.018	0.277 *	0.080 *	0.125 *	0.047 *	0.275 *
6-nearest neighbors	0.513 *	0.321 *	0.018	0.265 *	0.084 *	0.122 *	0.049 *	0.271 *
7-nearest neighbors	0.498 *	0.315 *	0.018	0.253 *	0.078 *	0.118 *	0.045 *	0.263 *
8-nearest neighbors	0.486 *	0.309 *	0.018	0.246 *	0.073 *	0.119 *	0.047 *	0.258 *
4km distance band	0.472 *	0.318 *	0.024	0.293 *	0.090 *	0.127 *	0.056 *	0.272 *

* Significant at the 0.05 level.

**Table 2.** Sample percent of local spatial pattern types of LISA analysis (%).

**Types of spatial autocorrelation**	**Cr**	**Ni**	**Zn**	**Hg**
No significance	56.09	69.94	66.70	67.78
High-high	14.34	7.07	8.74	9.63
Low-low	22.20	12.48	13.46	11.30
Low-high	3.05	7.96	7.56	8.35
High-low	4.32	2.55	3.54	2.95

**Table 3.** Sample pollution status distribution in local spatial pattern types (%).

**Heavy metals**	**Pollution status**	**Types of spatial autocorrelation**				
		**No significance**	**High-high**	**Low-low**	**Low-high**	**High-low**
Cr	Polluted		0.69			
	Unpolluted	56.09	13.65	22.20	3.05	4.32
Ni	Polluted	2.65	0.79		0.49	
	Unpolluted	67.29	6.29	12.48	7.47	2.55
Zn	Polluted	0.10				
	Unpolluted	66.60	8.74	13.46	7.56	3.54
Hg	Polluted	3.44	2.26		0.49	0.10
	Unpolluted	64.34	7.37	11.30	7.86	2.85
